# Type of Physical Training and Selected Aspects of Psychological Functioning of Women with Obesity: A Randomised Trial

**DOI:** 10.3390/nu13082555

**Published:** 2021-07-26

**Authors:** Monika Bąk-Sosnowska, Magdalena Gruszczyńska, Damian Skrypnik, Sławomir Grzegorczyn, Joanna Karolkiewicz, Marzena Ratajczak, Edyta Mądry, Jarosław Walkowiak, Paweł Bogdański

**Affiliations:** 1 Department of Psychology, Chair of Social Sciences and Humanities, School of Health Sciences in Katowice, Medical University of Silesia in Katowice, 40-055 Katowice, Poland; monika.bak-sosnowska@sum.edu.pl (M.B.-S.); mgruszczynska@sum.edu.pl (M.G.); 2 Department of Treatment of Obesity, Metabolic Disorders and Clinical Dietetics, Poznan University of Medical Sciences, 61-701 Poznań, Poland; pbogdanski@ump.edu.pl; 3 Department of Biophysics, School of Medicine in Zabrze, Medical University of Silesia in Katowice, 40-055 Katowice, Poland; grzegorczyn@sum.edu.pl; 4 Department of Food and Nutrition, Poznan University of Physical Education, 61-701 Poznań, Poland; karolkiewicz@awf.poznan.pl; 5 Department of Anatomy, Poznan University of Physical Education, 61-701 Poznań, Poland; mratajczak@awf.poznan.pl; 6 Department of Physiology, Poznan University of Medical Sciences, 61-701 Poznań, Poland; edytamadry@gmail.com; 7 Department of Paediatric Gastroenterology and Metabolic Diseases, Poznan University of Medical Sciences, 61-701 Poznań, Poland; jarwalk@ump.edu.pl

**Keywords:** obesity, physical activity, psychological aspects, body image

## Abstract

Objective: We conducted a prospective randomised trial to assess whether a specific type of regular physical training performed by women with obesity is related to obtaining specific psychological benefits. Methods: Forty-four women qualified for the study and were divided into two groups. The applied intervention consisted of regular three-month physical exercises in the form of endurance training (group A) or endurance strength training (group B). Initially, and after the completed intervention, we examined anthropometric measurements and the level of: stress (PSS-10), general self-esteem (SES), body self-report (BSQ–34, FRS), and behaviours associated with diet (TFEQ-18). Results: As a result of the intervention, both groups had significantly lower anthropometric parameters and FRS scores with regard to the current figure (gr. A:δ FRS CS −0.90 ± 0.83, *p* < 0.001; gr. B:δ FRS CS −0.41 ± 0.50, *p* = 0.01) and BSQ–34 results (gr. A:δ BSQ–34 −14.90 ± 13.5, *p* = 0.001; gr. B:δ BSQ–34 − 18.64 ± 25.4, *p* = 0.01). Additionally, an increase in cognitive restraint (δ TFEQ–18 CR1.65 ± 2.06, *p* = 0.01) and a decrease in emotional eating (δ TFEQ–18 EE −0.82 ± 1.28, *p* = 0.01) were observed in group B. There were no between-group differences in terms of the magnitude of changes achieved due to the intervention, except for asignificant improvement in the perception of their current figure (FRS) (δ FRSCS −0.90 ± 0.83, *p* = 0.03) in group A. Conclusions: Regular physical activity over a three-month period by women with obesity promotes the perception of their own body as slimmer and lowers body shape concerns. The change in body shape perception was more pronounced under the influence of endurance training than endurance strength training. Trial registration: ClinicalTrials.gov ID NCT04793451.

## 1. Introduction

Physical activity is recommended as one of the key methods of reducing excess body weight, in addition to caloric restriction. It helps to improve control of type 2 diabetes (T2D) [1] and cardiovascular diseases (CVD) [2], reduces serum glucose levels and blood pressure, improves overall quality of life [3], reduces insulin resistance [4], serum levels of proinflammatory cytokines [5] and the risk of cancer [6], and helps to maintain physical fitness [7]. The benefits of physical activity are observed in people with obesity even when they do not significantly reduce their body weight, as maintaining a high level of cardiorespiratory fitness reduces the risk of obesity-related diseases [8]. Additionally, it helps older patients to maintain the appropriate telomere length as an indicator of cellular biological ageing [9]. However, this phenomenon applies to regular patterns of high physical activity but not occasional physical activity, which is more strongly associated with a number of anthropometric and mortality outcomes [10]. Some studies show that, although physical activity brings measurable health benefits regardless of gender, positive health outcomes can be observed in women even for low (<3 MET) or moderate (3–6 MET) intensity exercise performed for 15 min a day [11].

In the case of overweight people (BMI 25–29.9 kg/m^2^), 45 to 60 min of moderate physical activity most days a week is recommended, while for people with obesity (BMI ≥ 30 kg/m^2^), the recommended amount is 60 to 90 min [12]. It is estimated that regular aerobic physical activity for less than 150 min a week may only prevent further weight gain, but slight weight loss (2–3 kg) requires 150–225 min of physical activity a week. Weight reduction at the level of 5–7.5 kg may be achieved by regular physical activity for more than 225 min (to 420 min) weekly. In order to maintain the achieved results, moderate physical activity for 200–300 min a week is recommended [13].

Endurance training is recommended for people with obesity; however, it brings satisfactory results (at the level of a 5–15% reduction ininitial body weight) only in combination with caloric restriction [14]. Even if it does not bring about significant weight loss, it improves glucose and lipid metabolism in people who previously led a sedentary lifestyle [15]. In turn, strength training is considered ineffective in reducing excess body weight. However, it should be noted that it brings measurable health benefits, such as a reduction inadipose tissue, increased muscle mass and an improvement in the lipid profile [16]. In women with abdominal obesity who performed endurance training or mixed endurance strength training three times a week for three months, some beneficial effects were observed in terms of anthropometric parameters, body composition, physical fitness and cardiovascular function [17], liver [18] and kidney parameters [19], as well as mineral balance [20], functional skills and back pain [21].

Physical activity is beneficial both for somatic functions and mental health [22], as well asforthe efficiency of psychological mechanisms, even in terms of motivation, self-efficacy or self-regulation [9]. This is especially important in people with obesity, as the disease is often accompanied by depression, decreased quality of life and self-esteem, appearance-related embarrassment, withdrawal from social relationships and other psychological problems [23]. The relationship between the perception of one’s own appearance and self-esteem, overall mental wellbeing and eating behaviour is important, especially in women [24,25,26]. Scientific reports confirm the psychological benefits of endurance training in people with excess body weight, which can lead to lower levels of depression, improved moods and overall self-esteem [27]. Similar effects can be brought about by less intensive Pilates training [28] or yoga [29]. On the other hand, a meta-analysis comparing the results of 17 studies on the effect of strength training on the mental status of people with obesity did not show any convincing evidence of such an effect. A small effect, however, was observed with regard to aspects such as self-esteem, inhibition, anxiety or depression [30].

A comparison of psychological benefits resulting from both types of physical training in a group of people with obesity has barely been studied to date. The available results are mainly focusedon the level of depression, anxiety and quality of life and indicate no significant differences between groups performing endurance and endurance strength training [31,32]. Therefore, the aim of our study was to compare the influence of endurance and endurance strength training on stress, self-esteem, body-esteem and eating behaviour in women with obesity. The innovative nature of the study is related to the use of acomparative intervention model (endurance vs. endurance strength) and analysis of the impact of these two training models on the psychological state of women with obesity.

## 2. Materials and Methods

### 2.1. Trial Information

The study was a part of an interdisciplinary research project for which consent from the Bioethics Committee of the Medical University in Poznań was obtained (No. 1077/12 with supplement No. 753/13). The study took place between December 2012 and December 2017.

This work was supported by the National Science Centre, Poland, Grant No. 2014/13/B/NZ7/02209 and the Faculty of Medicine I, Poznan University of Medical Sciences, Poland, Grant for Young Scientists [grant number 502-14-01119172-41123].

The study has been registered as a clinical trial on ClinicalTrials.gov under the ID NCT04793451. The trial protocol can be accessed at https://clinicaltrials.gov/ct2/show/NCT04793451 (accessed on: 22 July 2021).

### 2.2. Participants

The screening was conducted among 163 people registered in the outpatient clinic of the Department of Internal Medicine, Metabolic Disorders, and Hypertension, University of Medical Sciences, Poznan, Poland. Inclusion criteria relevant to the issues presented in this study were as follows: (1) informed and written consent to participate in the study, (2) age between 18 and 65, (3) diagnosed obesity (BMI ≥ 30 kg/m^2^), (4) stable body weight for a month before the study (acceptable deviation ± 1 kg). Exclusion criteria relevant for this paper were as follows: (1) intellectual disability, (2) mental illness, either currently or in medical records, (3) pregnancy, childbirth and lactation. The full list of criteria is presented in a publication by Skrypniket et al. [17]. A total of 44 people qualified for the study.

### 2.3. Study Design

The study was a prospective randomised trial. The study group was divided into two subgroups (group A and group B) using a randomisation list. Patient enrolment was undertaken by the physician. Patients were assigned to the study intervention by the physician using the subject’s unique code according to the random allocation sequence generated by a computer. The groups did not differ in age, body mass, BMI, waist and hip circumference, or WHR [17]. Both groups participated in parallel physical training for three months. Group A (*n* = 22) took part in endurance training, while group B (*n* = 22) took part in endurance strength training. The subjects were informed of the need to keep their diet unchanged. Based on a dietary interview before and after the intervention and with the use of the specialised software Diet 2.0 for product consumption analysis, it was found that nutrient intake, total calories and caffeine intake during the study in both groups were at a constant, comparable level.

Initially and after three months of physical training, measurements of body weight, height, waist and hips were conducted [17]. The following psychological parameters were also assessed: stress level, overall self-esteem, body self-esteem and eating behaviour. The study was conducted by means of adiagnostic survey method using standardised questionnaires.

The Perceived Stress Scale (PSS-10) by Cohen, Kamarcki and Mermelstein, adapted in Polish by Juczyński and Ogińska-Bulik [33] examines general stress levels. It contains 10 questions concerning various subjective feelings associated with problems and personal experiences, behaviours and ways of dealing with them. The respondent marks the answers on a 4-point scale (0, never; 1, hardly ever; 2, sometimes; 3, quite often; 4, very often). The total score is the sum of all points, and the higher the score, the higher the examined person’s stress intensity. The total score, after conversion in accordance with the guidelines of the authors into standardised units, is interpreted as low (1–4 sten), average (5–6 sten) or high (7–10 sten). The Cronbach’s alpha in our study was 0.81 before and 0.84 after the intervention.

The Rosenberg Self-Esteem Scale (SES) by Rosenberg, adapted in Polish by Dzwonkowska, Lachowicz-Tabaczek and Łaguna [34], examines levels of overall self-esteem. It is composed of 10 diagnostic statements to which respondents refer on a 4-point scale (1, I definitely agree; 2, I agree; 3, I have no opinion; 4, I do not agree; 5, I definitely do not agree). The final score is the sum of all points, and the higher the score, the higher the level of self-esteem of the examined person. The total score can be converted into stenunits and interpreted analogously in accordance with the guidelines of the authors, as described above. The Cronbach’s alpha in our study was 0.82 before and 0.80 after the intervention.

The Body Shape Questionnaire (BSQ-34) by Cooper, Taylor, Cooper and Fairburn [35] examines the level of one’s preoccupation with their own body shape. It is composed of 34 questions. Each question has a 6-point scale of answers thatreflects the frequency of experiencing the described situations by the subject in the last 30 days (1, never; 2, rarely; 3, sometimes; 4, often; 5, very often; 6, always). The sum of points gives the total score, making it possible to classify the examined person, in accordance with the authors’ guidelines, in one of four categories describing the level of concern with their body shape: none, mild, moderate and considerable. The Cronbach’s alpha in our study was0.94 before and 0.95 after the intervention.

The Figure Rating Scale (FRS) by Stunkart et al. [36] examines the perception of the size and shape of one’s own body. It presents nine schematic silhouettes of men and women, from extremely thin to extremely obese. Subjects are asked to choose the silhouette that best reflects their current silhouette (CS) and ideal silhouette (IS) physical appearance. In the presented study, only female silhouettes were used due to the specificity of the study group.

The Three-Factor Eating Questionnaire-18 (TFEQ-18) by Karlsson and Persson, adapted in Polish by Brytek-Matera, Rogoza and Czepczor-Bernat [37] is used for measuring eating behaviour. The subject responds to the questionnaire using a 4-grade scale (0, definitely not; 1, rather not; 2, rather yes; 3, definitely yes). The scores are calculated separately for three sub-scales: cognitive restraint of eating (CR), uncontrolled eating (UE) and emotional eating (EE). The higher the score achieved in the given sub-scale, the greater the intensity of the tested behaviour is manifested. The average Cronbach’s alpha for all sub-scales in our study was 0.71 before and 0.76 after the intervention.

The occurrence of any exclusion criteria (mentioned above) during the trial resulted in immediate cessation of participation in the study. There were no important changes to the methodology after the commencement of the trial. There were no changes to trial outcomes after the trial commenced.

### 2.4. Intervention

The intervention was planned for three months. During this time, the women participated in physical training three times a week. Each group had 36 training sessions. Training took place at a professional sports club, Sport Club City Zen in Poznań, and was conducted by a qualified and certified fitness instructor. Both groups were also under medical supervision. Training for both groups included the following exercises.

Group A: Endurance training on bicycle ergometers (Schwinn Evolution, Schwinn Bicycle Company, Boulder, CO, USA). Training sessions consisted of five minutes of warm-up, including stretching exercises of low intensity (50–60% of maximum heart rate), 45 min of training with an intensity of between 50 and 80% of maximum heart rate, five minutes of riding a cycloergometer without any additional load and five minutes of low-intensity stretching and breathing exercises.

Group B: Endurancestrength training that consisted of a five-minute warm-up (stretching exercises) of low intensity (50–60% of maximum heart rate), 20 min of strength exercises, 25 min of endurance exercises (50–80% of maximum heart rate), five minutes of cycling without any additional load and five minutes of stretching and breathing exercises.

As part of the strength exercises, the participants undertook some variable, repeated weekly exercises with a barbell and exercises with a gym ball. Endurance exercises were performedon a cycloergometer (Schwinn Evolution, Schwinn Bicycle Company, Boulder, CO, USA). Participants’heart rate during training was monitored using a Suunto Fitness Solution^®^ (Suunto, Vantaa, Finland) device. Both types of training were comparable in exercise volume and varied only in the modality of the effort. The intervention ended when the last participant completed the training. The exact description of the intervention is presented in the publication by Skrypniket et al. [17].

### 2.5. Statistical Analysis

The Statistica13.1 statistical package was used. The normality of distributions was tested using a Shapiro–Wilk test. Statistical methods were used to compare groups for primary and secondary outcomes (in the area of analysed variables, e.g., the level of stress and self-esteem, body perception and eating behaviour), as well as for subgroup analysis (e.g., the difference between the initial and end value of variables, correlations between variables). The difference between the initial and end value (delta-δ) was calculated for each analysed variable. In addition, differences between the groups that implemented different forms of physical training were examined with regard to the size of change (δ) in the analysed variables. The relationships between changes of the studied parameters (delta results) were investigated using the correlation coefficient.

Parametric tests (t-Student tests for dependent and independent variables) were used for the analysis of variables with normal distribution, while nonparametric tests (a Wilcoxon signed-rank test for dependent variables and a Mann–Whitney U test for independent variables) were used in the case of a lack of normality in variables distribution. Differences in categorical variables were compared with Fisher’s exact test. The strength of the relationship between variables was calculated using d-Cohen’s effect size (for dependent variables) and g-Hedges’ effect size (for independent variables). The adopted level of significance was α < 0.05.

## 3. Results

**Figure 1:**
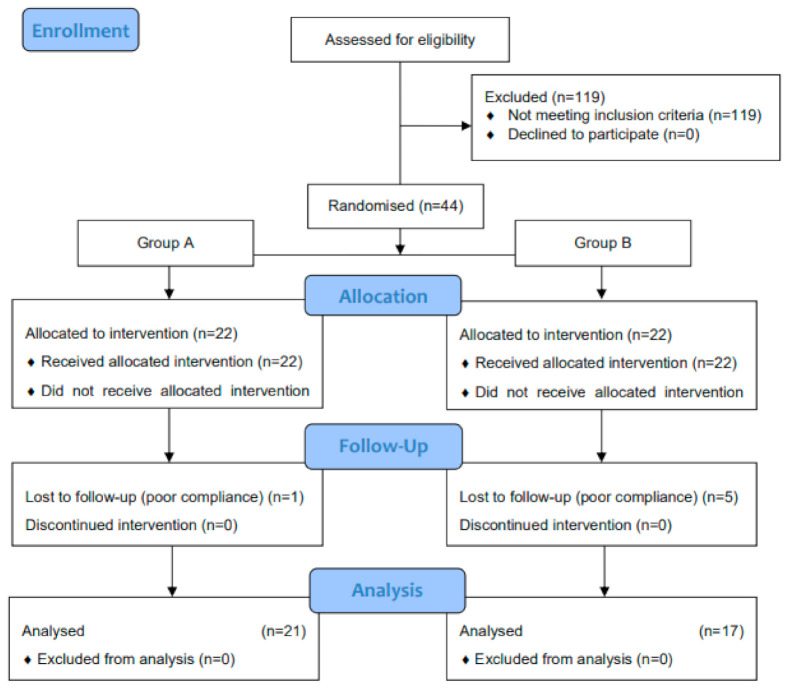
Flow diagram of study groups.

During the intervention, six subjects, one from group A and five from group B, were withdrawn from the trial due to poor compliance, defined as attendance at fewer than 60% of physical training sessions. In the endurance training group, the average attendance at classes was 81.97%, while in the endurance and strength training group, it was 87.37%. There was no statistically significant difference between the groups of women in terms of attendance at classes (*p* = 0.1652). Thirty-eight patients (group A, *n* = 21; group B, *n* = 17) completed the study and underwent statistical analysis (Figure 1). The analysis was performed by the original assigned groups. No important harm or unintended effects in each group occurred.

Comparison of studied parameters between groups A and B before and after the intervention was performed. The results of the comparison are presented in Table 1.

In addition, in terms of nutrient, calorie and caffeine intake, there was no significant difference between groups A and B before and after the intervention (Table S1).

Before the intervention, groups A and B did not differ significantly in terms of any analysed variable. After the intervention, the only significant difference was emotional eating. In the endurance strength training group, as well as before the intervention, the level of this variable was higher than in the endurance training group. In the endurance training group, the average attendance at classes was 81.97%, while in the endurance and strength group, it was 87.37%. There was no statistically significant difference between the groups in terms of attendance at classes. The Mann–Whitney U test was *p* = 0.1652.

As a result of the intervention used, significant changes in anthropometric parameters were observed in both groups, which have already been presented in the paper by Skrypnik et al. [17]. With regard to psychological parameters after the intervention, there was a perception of the current figure as significantly slimmer and a significant reduction in level body shape concerns in both groups. Additionally, eating behaviour improved in group B, namely, the level of cognitive restraint increased, and emotional eating level decreased. Details are presented in the table below (Table 2).

**Figure 2:**
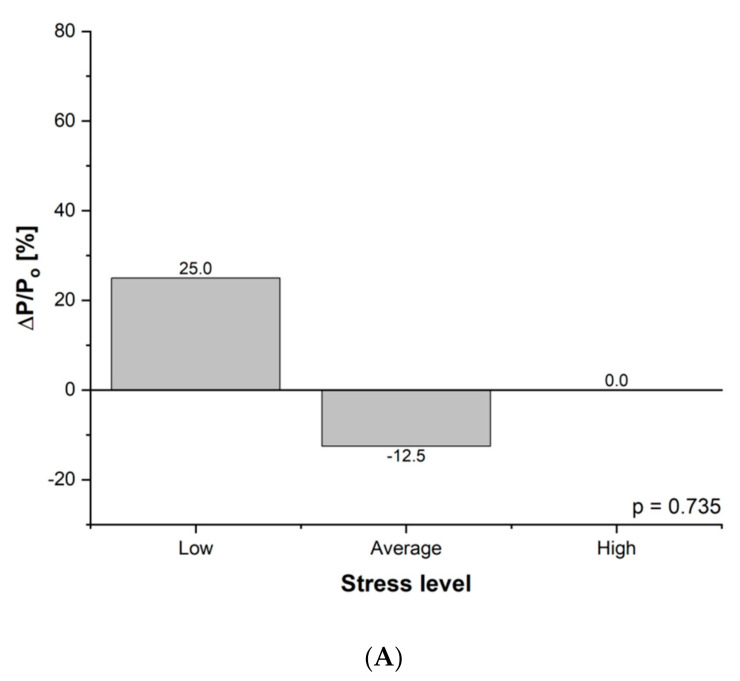

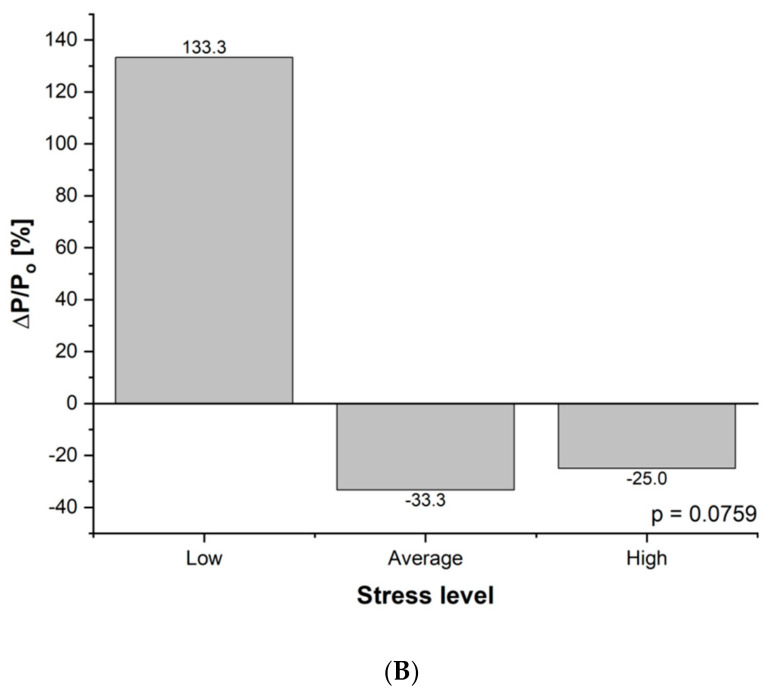
(**A**) Difference in stress level initially and after three months of intervention in group A: P, percentage of the group; ΔP = P_After_ − P_before_; P_o_ = P_before_; ΔP/P_o_ relative change of percentage of the group (during experiment). (**B**) Difference in stress level initially and after three months of intervention in group B: P, percentage of the group; ΔP = P_After_ − P_before_; P_o_ = P_before_; ΔP/P_o_ relative change of percentage of the group (during experiment).

The number of people experiencing lowstress levels increased, and the number of people experiencing average stress levels decreased in both groups. Moreover, the number of people experiencing highstress levels decreased in group B (Figure 2A,B). However, the distribution of results did not change significantly.

**Figure 3:**
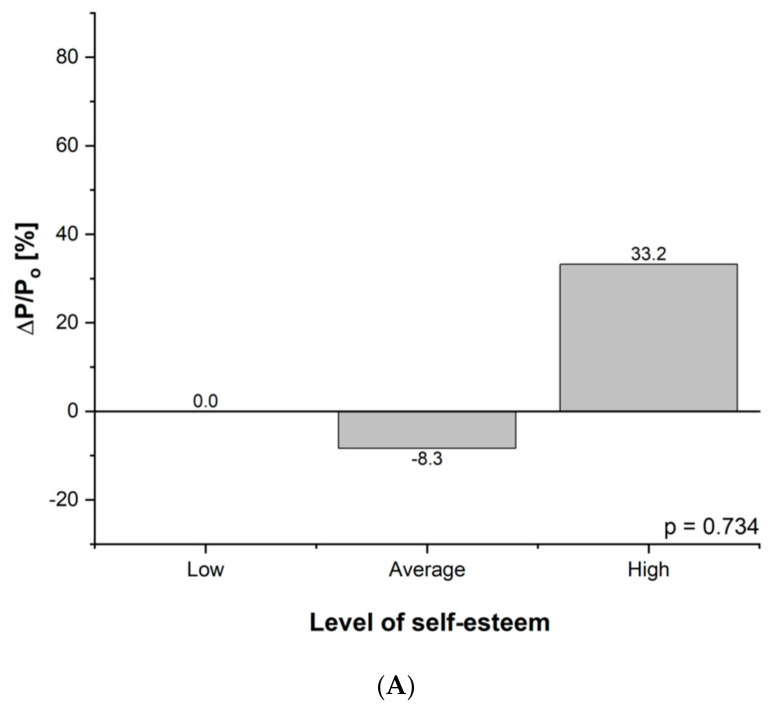

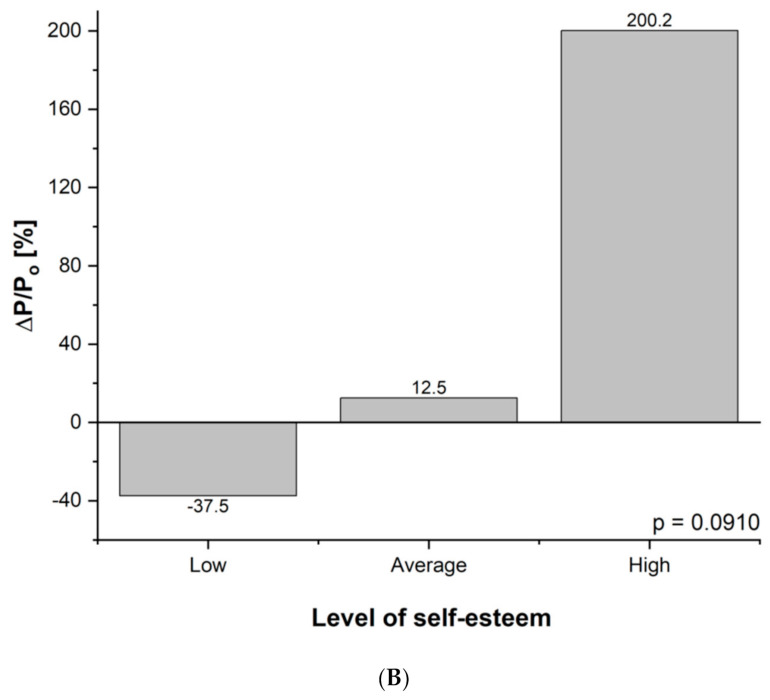
(**A**) Difference in self-esteem level initially and after three months of intervention in group A: P, percentage of the group; ΔP = P_After_ − P_before_; P_o_ = P_before_; ΔP/P_o_ relative change of percentage of the group (during experiment). (**B**) Difference in self-esteem level initially and after three months of intervention in group B: P, percentage of the group; ΔP = P_After_ − P_before_; P_o_ = P_before_; ΔP/P_o_ relative change of percentage of the group (during experiment).

In the case of general self-esteem, the number of people with high self-esteem increased in both groups. In the categories of low and average self-esteem, changes were clearer and potentially beneficial in group B (Figure 3A,B). However, the distribution of results did not change significantly.

As a result of the intervention, it was observed that as the level of self-esteem (δ SES) increased among the subjects, there were larger reductions in the following: BMI (r = −0.438), waist circumference (r = −0.349), EE (r = −0.330) and BSQ–24 (r = −0.377). Moreover, as the level of body shape concerns (δ BSQ–24) decreased, waist circumference (r = 0.393), WHR (r = 0.379), UE (r = 0.446) and CS (r = 0.362) also decreased. Additionally, the greater the difference in perception of the current silhouette of subjects (δ CS), the greater was the change in BMI (r = 0.375).

In addition, analysis of the variables before and after the intervention showed that only the size of the change in the perception of the current silhouette differentiated both groups in favour of group A. Details concerning all the analysed variables are presented in Table 3.

## 4. Discussion

After three months of regular physical training, all subjects, regardless of the type of activity, showed significant changes in the objective parameters. A decrease in body weight, waist and hips circumference, as well as BMI and WHR, was observed. Certainly, this can be a reason for a better perception of the current figure and lowering of the level of concern about one’sbody shape. The importance of BMI and WHR for the mental wellbeing of women is due to the fact that both parameters are related to physical health [38]. The researchers didnot agree on which of them is more important for the assessment of the attractiveness of the female figure. For instance, Furnham et al. [39] studied different body models of women with different body weights, WHRs and breast sizes. They assessed the influence of certain patterns on the assessment of women’s attractiveness, femininity, health and fertility. They observed the strongest effect for WHR (0.34–0.52) but not for body weight (0.14–0.31). In the case of breast size, no major effect was shown [39]. In line with this observation, Płatek and Sighn [40] confirmed the importance of optimal (approx. 0.7) WHR in women’s attractiveness. By contrast, Holliday et al. [41] showed that BMI, not WHR, modulates reward mechanisms in the brain in both men and women.

Despite these differences, it was clearly confirmed that both body shape improvement (expressed as a decrease in WHR) and weight loss (corresponding to BMI reduction) in women with obesity have a positive impact on the level of satisfaction with their own body and reduce concerns about their appearance [42,43]. Even though we did not prove a significant improvement in the self-esteem of the subjects in our study, we did observe a tendency for its improvement, especially in the group performing endurance and strength training. The positive effect of excess weight reduction on self-esteem was also observed by the other authors [44]. Interestingly, most women show dissatisfaction with their own body regardless of their actual body weight, and the level of dissatisfaction is higher in women with overweight and obesity [45]. In women, dissatisfaction with one’s appearance and worrying about one’s figure has a strong negative effect on their overall sense of self-respect and on behaviour, as well as on nutrition [46]. From a psychological point of view, certain manifestations of eating behaviour may indicate risk or the occurrence of eating disorders. Common eating behaviours include, for example, cognitive food abstention, such as intentionally abstaining from eating or limiting food intake in order to control both body weight and body image. In turn, uncontrolled eating manifests itself in a tendency to overeat due to unrestrained hunger. Emotional eating is characterised by overeating due to depressed mood and anxiety. In our study, we confirmed the link between the severity of body shape concerns and uncontrolled eating. The more the level of body shape concerns decreased, the more the level of uncontrolled eating decreased.

Additionally, we have shown that the eating behaviour of women with excess body weight may be influenced by participation in a specific type of physical training. Namely, after three months’ intervention, the endurance and strength training group had significantly higher levels of cognitive restraint and significantly lower levels of emotional eating compared with the endurance training group. The observed differences may be the result of the relationship between eating behaviours and the level of stress, which in the group with endurance and strength training decreased more significantly as a result of the intervention; however, the difference between groups was not statistically significant. Lower levels of stress improve cognitive functioning, including cognitive behavioural control and goal achievement [47]. It also promotes the effective regulation of emotions, which reduces emotional eating and unfavourable food choices [48]. Although physical training generally helps reduce stress, the fact that the specificity of training and the presence of such elements as breathing and stretching exercises, which takes place in endurance and strength training, are also important and cannot be ruled out. We observed this tendency in our study comparing endurance training versus endurance and strength training, but this hypothesis needs to be tested on a larger population.

Although changes in the intensity of stress experienced by women participating in the intervention turned out to be statistically insignificant, we observed a clear trend related to the increase in the number of people experiencing low levels of stress and a tendency towards more favourable changes in this regard in women performing endurance and strength training. The relationship between obesity and stress at both the cellular level (oxidative stress) and psychological and social levels have been already confirmed in other studies [49,50]. The lack of clear changes in stress intensity during the intervention may be related to the assessment of the results obtained as lower than expected by the subjects. However, the effort put into regular physical activity resulted in are duction of objective somatic parameters, but in the case of high initial body weight, the changes may be less noticeable both for the examined subject and for her social environment. Another reason may be the presence of a mediating factor between the change in appearance and appearance-related stress. The potential level of self-esteem, the level of self-compassion or the level of depression can be such a factor. However, these hypotheses need to be confirmed in further studies.

The main purpose of our study was to specify whether the size of changes in selected psychological variables differs depending on the form of physical training. Endurance training develops features such as agility, speed, flexibility and nimbleness. It involves all muscle groups and improves the functioning of the heart and entire circulatory system [51]. Endurance and strength training is additionally completed with resistance exercises aimed at increasing both muscle and bone strength and improving metabolism [52]. We did not observe any significant differences in the size of psychological changes, except for the assessment of one’s current body shape, among the subjects who followed the abovementioned forms of training for three months. Although self-esteem related to the current figure changed with training towards a slimmer silhouette in both groups, this change was significant only in women who undertook endurance training.

### 4.1. Strengths of the Study

No similar studies have been found in the scientific literature that could be used as a comparison. It is only known that both types of training bring similar improvements in depression and anxiety as well as quality of life in people with excess body weight [29,30].

In our study, we used an innovative, comparative model of an endurance and endurance strength training intervention to analyse the impact of certain forms of physical activity on the psychological functioning of women with obesity. We applied strict inclusion and exclusion criteria, which resulted in a homogeneous group of participants. We have proven that both forms of training improve body image parameters, such as body perception and body concerns. In addition, we found that endurance strength training significantly improved two psychological parameters influencing eating behaviour: emotional eating and cognitive restraint, and that this form of physical activity promoted better stress management. Our findings could play a potential role in creating recommendations for future obesity treatment, as they proved that endurance and strength training brings some psychological benefits in addition to measurable physiological changes.

### 4.2. Study Limitations

Our results should be considered preliminary and requiring confirmation in a study intentionally designed to assess the changes in body image and eating behaviour under the influence of a specific type of physical activity. Further studies will also bean opportunity to correct the shortcomings that we could not avoid in this article. The basic limitation of the study is the relatively small number of participants and the significant age range.

## 5. Conclusions

Three months of regular physical activity in women with obesity promotes the perception of their own body as thinner, and it reduces concerns about their body shape. The change in body shape perception was more pronounced with endurance training compared with endurance strength training.

## Figures and Tables

**Table 1:** Comparison of studied parameters between groups A and B before and after the intervention (anthropometric parameters have already been presented in the paper by Skrypnik et al. 2015 [17]).

Variables		Group A before Intervention (*n* = 21)	Group B before Intervention (*n* = 17)	*p*	g	Group A after Intervention (*n* = 21)	Group B after Intervention (*n* = 17)	*p*	g
Body mass [kg]		91.7 ± 11.8	94.5 ± 13.4	0.325	0.2234	89.5 ± 11.8	91.8 ± 13.7	0.628	0.1814
BMI [kg/m^2^]		35.2 ± 3.9	34.9 ± 3.8	0.747	0.0778	34.3 ± 3.9	33.9 ± 4.1	0.725	0.1002
Waist circumference [cm]		110.8 ± 10.2	111.6 ± 11.3	0.883	0.0747	105.5 ± 11.1	104.0 ± 10.5	0.538	0.1384
Hip circumference [cm]		115.0 ± 8.0	115.8 ± 9.4	0.577	0.0924	111.7 ±8.5	112.4 ±9.7	0.837	0.0773
WHR		0.96 ± 0.06	0.96 ± 0.07	0.770	0.00	0.94 ± 0.07	0.92 ± 0.07	0.445	0.2857
PSS-10		18.00 ± 7.3	18.8 ± 7.4	0.714	0.1089	17.1 ± 7.2	17.2 ± 7.3	0.973	0.0138
SES		29.1 ± 4.6	27.2 ± 4.1	0.183 *	0.4333	29.4 ± 4.09	28.3 ± 4.3	0.414	0.2629
BSQ-34		97.4 ± 23.07	99.2 ± 30.2	0.836	0.0680	82.5 ± 24.4	80.9 ± 25.8	0.814	0.0639
FRS	CS	6.62 ± 0.92	6.70 ± 1.16	0.978 *	0.0774	5.71 ±0.78	6.29 ±1.31	0.230 *	0.5528
	IS	4.04 ± 0.59	4.12 ± 0.60	0.726 *	0.1346	4.00 ± 0.63	4.00 ± 0.71	0.987 *	0.00
TFEQ-18	CR	14.5 ±3.1	13.8 ± 2.8	0.483 *	0.2357	14.7 ± 2.5	15.5 ± 2.6	0.371	0.3144
	UE	20.4 ±3.8	20.5 ± 4.6	0.948	0.0239	19.3 ±3.7	19.2 ± 4.4	0.906	0.0248
	EE	6.8 ± 2.5	8.4 ± 2.09	0.0835 *	06877	6.4 ± 1.4	7.6 ± 2.001	**0.037**	0.7087

BMI, body mass index; CS, current silhouette; IS, ideal silhouette; CR, cognitive restraint; UE, uncontrolled eating; EE, emotional eating; *p*, level of statistical significance; g, Hedges’ effect size; * Mann–Whitney U test. Significant *p* value is pointed bold.

**Table 2:** Comparison of studied parameters before and after the intervention in groups A and B (anthropometric parameters have already been presented in the paper by Skrypnik et al. 2015 [17]).

Variables		Group A before Intervention (*n* = 21)	Group A after Intervention (*n* = 21)	*p*	d	Group B before Intervention (*n* = 17)	Group B after Intervention (*n* = 17)	*p*	d
Body mass [kg]		91.7 ± 11.8	89.5 ± 11.8	**<0.001**	0.181	94.5 ± 13.4	91.8 ± 13.7	**0.003**	0.193
BMI [kg/m^2^]		35.2 ± 3.9	34.3 ± 3.9	**<0.001**	0.225	34.9 ± 3.8	33.9 ± 4.1	**<0.001**	0.245
Waist circumference [cm]		110.8 ± 10.2	105.5 ± 11.1	**<0.001**	0.485	111.6 ± 11.3	104.0 ± 10.5	**<0.001**	0.675
Hip circumference [cm]		115.0 ± 8.0	111.7 ± 8.5	**<0.001**	0.390	115.8 ± 9.4	112.4 ±9.7	**0.001**	0.345
WHR		0.96 ± 0.06	0.94 ± 0.07	**0.010**	0.299	0.96 ± 0.07	0.92 ± 0.07	**0.005**	0.554
PSS-10		18.00 ± 7.3	17.1 ± 7.2	0.471	0.121	18.8 ± 7.4	17.2 ± 7.3	0.151	0.211
SES		29.1 ± 4.6	29.4 ± 4.09	0.298	0.067	27.2 ± 4.1	28.3 ± 4.3	0.106 *	0.254
BSQ-34		97.4 ± 23.07	82.5 ± 24.4	**<0.001**	0.612	99.2 ± 30.2	80.9 ± 25.8	**0.008**	0.632
FRS	CS	6.62 ± 0.92	5.71 ±0.78	0.001 *	1.041	6.70 ± 1.16	6.29 ±1.31	**0.017**	0.321
	IS	4.04 ± 0.59	4.00 ± 0.63	0.767 *	0.063	4.12 ± 0.60	4.00 ± 0.71	0.361 *	0.177
TFEQ-18	CR	14.5 ±3.1	14.7 ± 2.5	0.802 *	0.069	13.8 ± 2.8	15.5 ± 2.6	**0.004**	0.610
	UE	20.4 ±3.8	19.3 ±3.7	0.233	0.286	20.5 ± 4.6	19.2 ± 4.4	0.135	0.280
	EE	6.8 ± 2.5	6.4 ± 1.4	0.394 *	0.192	8.4 ± 2.09	7.6 ± 2.001	**0.017**	0.379

BMI, body mass index; CS, current silhouette; IS, ideal silhouette; CR, cognitive restraint; UE, uncontrolled eating; EE, emotional eating; *p*, level of statistical significance; d, Cohen’s effect size; * Wilcoxon sign test. Significant *p* value is pointed bold.

**Table 3:** Comparison of change in the parameters studied from the baseline to the three-month point of the intervention in groups A and B (anthropometric parameters have already been presented in the paper by Skrypnik et al. 2015 [17]).

Variables		Group A (*n* = 21)	Group B (*n* = 17)	*p*	g
δ Body mass [kg]		−2.20 ± 2.12	−2.71 ± 2.25	0.371	0.234
δ BMI [kg/m^2^]		−0.84 ± 0.80	−0.99 ± 0.80	0.348	0.187
δ Waist circumference [cm]		−5.26 ± 4.45	−7.65 ± 4.56	0.142 *	0.531
δ Hip circumference [cm]		−3.33 ± 2.83	−3.41 ± 3.58	0.547	0.025
δ WHR		−0.02 ± 0.03	−0.04 ± 0.05	0.207	0.498
δ PSS-10		−0.90 ± 5.64	−1.71 ± 4.66	0.642	0.154
δ SES		0.33 ± 2.43	1.12 ± 2.61	0.447 *	0.314
δ BSQ–34		−14.90 ± 13.5	−18.64 ± 25.4	0.565	0.189
δ FRS	CS	−0.90 ± 0.83	−0.41 ± 0.50	0.035 *	0.697
	IS	−0.05 ± 0.67	−0.12 ± 0.48	0.766 *	0.118
δ TFEQ–18	CR	0.24 ± 2.96	1.65 ± 2.06	0.106	0.542
	UE	−1.05 ± 3.90	−1.29 ± 3.38	0.538 *	0.065
	EE	−0.43 ± 2.09	−0.82 ± 1.28	0.941 *	0.219

BMI, body mass index; CS, current silhouette; IS, ideal silhouette; CR, cognitive restraint; UE, uncontrolled eating; EE, emotional eating; *p*, level of statistical significance; g, Hedges’ effect size; * Mann–Whitney U test. Significant *p* value is pointed bold.

## Data Availability

The data are stored on a secured research server of Statistics Poland and are accessible only to authorised researchers based on collaborative agreements. Please contact Monika Bąk-Sosnowska (monika.bak-sosnowska@sum.edu.pl) for further details.

## Supplementary Materials

The following are available online at https://www.mdpi.com/article/10.3390/nu13082555/s1, Supplementary Table S1: dietary intake of calorie, protein, carbohydrates, fat and caffeine in both the study groups before and after the intervention.
