# Supplementary material for: Type of Physical Training and Selected Aspects of Psychological Functioning of Women with Obesity: A Randomised Trial
Source: Nutrients. 2021 Jul 26;13(8):2555. doi: 10.3390/nu13082555 (PMC8400574; doi:10.3390/nu13082555)
Supplement: Supplementary file 1 [file nutrients-13-02555-s001.zip › nutrients-1256852-supplementary.pdf]

Supplementary table 1: Dietary intake of calorie, protein, carbohydrates, fat and caffeine in both study groups before and after the intervention.

Variables	Group A before intervention	Group B before intervention	p^b	Group A after intervention	Group B after intervention	p^b
**Protein intake [g/day]**	78.82±19.80	64.75±13.81	0.0771	69.58±15.06	67.41±14.92	0.8357
**p^a**				0.4485	0.6043	
**Carbohydrates intake [g/day]**	237.46±73.51	223.75±40.84	0.6086	201.01±88.62	208.07±35.63	0.8749
				0.0983	0.0666	
**Fat intake [g/day]**	70.06±24.42	63.45±12.60	0.4528	56.97±29.44	55.25±14.15	0.9107
**p^a**				0.0627	0.7513	
**Caffeine intake [mg/day]**	157.56±81.85	88.79±65.59	0.0528	151.12±45.46	91.20±82.70	0.2380
**p^a**				0.1904	0.3913	
**Energy [kcal/day]**	1895.64±530.58	1725.05±254.38	0.3674	1657.41±667.06	1611.78±243.50	0.8901
**p^a**				0.0981	0.1802	

[a]The paired sample t-test (within group over time differences)
[b]The independent samples t-test (between-group differences)
